# Improving the filtering of false positive single nucleotide variations by combining genomic features with quality metrics

**DOI:** 10.1093/bioinformatics/btad694

**Published:** 2023-11-29

**Authors:** Kazım Kıvanç Eren, Esra Çınar, Hamza U Karakurt, Arzucan Özgür

**Affiliations:** Department of Computer Engineering, Kocaeli University, Kocaeli 41000, Turkey; R&D Department, Idea Technology Solutions LLC., Istanbul 34396, Turkey; R&D Department, Idea Technology Solutions LLC., Istanbul 34396, Turkey; Department of Bioengineering, Gebze Technical University, Kocaeli 41400, Turkey; Department of Computer Engineering, Boğaziçi University, Istanbul 34342, Turkey

## Abstract

**Motivation:**

Technical errors in sequencing or bioinformatics steps and difficulties in alignment at some genomic sites result in false positive (FP) variants. Filtering based on quality metrics is a common method for detecting FP variants, but setting thresholds to reduce FP rates may reduce the number of true positive variants by overlooking the more complex relationships between features. The goal of this study is to develop a machine learning-based model for identifying FPs that integrates quality metrics with genomic features and with the feature interpretability property to provide insights into model results.

**Results:**

We propose a random forest-based model that utilizes genomic features to improve identification of FPs. Further examination of the features shows that the newly introduced features have an important impact on the prediction of variants misclassified by VEF, GATK-CNN, and GARFIELD, recently introduced FP detection systems. We applied cost-sensitive training to avoid errors in misclassification of true variants and developed a model that provides a robust mechanism against misclassification of true variants while increasing the prediction rate of FP variants. This model can be easily re-trained when factors such as experimental protocols might alter the FP distribution. In addition, it has an interpretability mechanism that allows users to understand the impact of features on the model’s predictions.

**Availability and implementation:**

The software implementation can be found at https://github.com/ideateknoloji/FPDetect.

## 1 Introduction

Next-Generation Sequencing (NGS) is extensively used by diagnostic laboratories to discover pathological variants for the diagnosis of suspected genetic disorders and in research to study the genetic architecture of diseases. Unfortunately, random or systematic errors in the sequencing, alignment and variant calling steps of NGS pipelines may result in incorrectly called variants [false positives (FPs)] or missed true variants, posing serious challenges for variant interpretation. The steady increase in the total number of candidate variants for clinical evaluation presents an additional challenge, as evidenced by the growth in databases such as OMIM and ClinVar (Amberger *et al.* 2015, Landrum *et al.* 2018).

FP errors can arise from sample preparation (e.g. sequencing reagents), the sequencing process (the hidden batch factors of the sequencing platform), and the preferences of the heuristic methods used in the analysis pipeline tools (and from the propagation of potential errors through the data analysis pipeline) (Luedtke *et al.* 2011, O’Fallon *et al.* 2013, Jun *et al.* 2015). Repeating genomic regions, such as tandem repeats, homopolymer regions, and segmental duplications are also very challenging for short-read data alignment. When mapping short-read data to a reference genome, read misalignment has been shown to be a major cause of incorrectly called variants (Li 2014).

To eliminate FPs, filtering tools such as Hard Filtering (HF) and Variant Quality Score Recalibration (VQSR) provided by the Genome Analysis Toolkit (GATK) (McKenna *et al.* 2010) are commonly used. However, these methods have some limitations. VQSR is highly dependent on user-selected parameters and fails to run in some cases [e.g. when Variant Call Format (VCF) files contain only a small number of variants]. In HF, variants are filtered based on certain thresholds chosen for a set of selected annotations. This approach is very limiting, since a single annotation can filter out a true variant even if all other annotations comply with the thresholds.

The variants identified by NGS can be confirmed either by performing orthogonal testing with Sanger sequencing or by comparing them with high-confidence truth datasets. Since orthogonal confirmation of all reported variants is costly, most studies that use this approach analyze a small number of variants from a small number of genes (Strom *et al.* 2014, Baudhuin *et al.* 2015, Mu *et al.* 2016, van den Akker *et al.* 2018). Lincoln *et al.* (2019) use a combination of truth sets along with orthogonally confirmed clinical test results. They manually select candidate thresholds that are converted into inputs for a heuristic algorithm that classifies variant calls as true positives or as calls requiring confirmation.

Due to the time-consuming and costly nature of orthogonal confirmation and the potential errors in threshold-based filtering, supervised machine learning-based approaches have been proposed as a promising solution, where the goal is to learn the probability distribution of annotations (features) of incorrect and correct variants from the data. van den Akker *et al.* (2018) propose a logistic regression based classifier to distinguish between true calls and those that require orthogonal confirmation. They use features related to the genomic position in which the call was made, such as GC content and proximity to homopolymers as well as quality metrics associated with variant calling. Other studies that use quality metrics extracted from VCF files include (Zhang and Ochoa 2020) and (Holt *et al.* 2021), which use methods such as Gradient Boosting, EasyEnsemble, Random Forest, and AdaBoost. Both studies use Genome in a Bottle Consortium (GIAB) truth sets and train separate models for insertion/deletion variants (Indels) and Single Nucleotide Variations (SNVs). Ravasio *et al.* (2018) use a deep learning model (a multi-layer perceptron algorithm) to classify variants in exome sequencing experiments performed with Illumina or ION Torrent platforms, using GATK quality metrics.

In this study, we approach FP detection as a supervised learning problem, using variant call data with known true variants (i.e. truth sets or gold standard/platinum standard data) for training and testing. To this end, we use exome data obtained from the National Center for Biotechnology Information Sequence Read Archive (NCBI-SRA) and compare our called variants with the high-confidence truth set characterized by the Genome in a Bottle (GIAB) Consortium (Zook *et al.* 2014, 2016, 2019). We analyze data with a pipeline using GATK Best Practices (McKenna *et al.* 2010), then classify variants as true or false using the gold/platinum standard data, and convert the annotations of each variant into features for the classification algorithm. Even though some studies propose modified variant calling pipelines and tools such as Pibase (Forster *et al.* 2013), GotCloud (Jun *et al.* 2015), SNPSVM (O’Fallon *et al.* 2013), and DeepVariant (Poplin *et al.* 2018), GATK is still the most commonly used variant calling pipeline. Our proposed method, once trained, can be directly applied to a VCF file, even to an annotated unique variant, and can be integrated into existing GATK-based analysis pipelines.

Our model is unique in that we use both genomic features of variants and GATK quality metrics to predict FP variants. Since some genomic regions are error-prone during alignment, we used genomic features of variants such as GC content and CpG islands. Other genomic features that we propose using for FP variant detection include the type of variation (transversion/transition), the type of amino acid replacement caused by a particular mutation, as well as features related to evolutionary conservation such as ancestral bases and information about whether the observed allele is evolutionary derived or not. Our experiments show that some of these features are more informative in distinguishing between true and FP variants than some features based on GATK metrics. Since, clinical NGS methods often emphasize sensitivity to avoid missing important variants, our models can be adjusted to allow application of different thresholds based on the required sensitivity (ability of a model to find true variants). We used data from Human sample NA12878 for training and initial testing. We also tested our models on another truth set data for an independent, nonrelative sample NA12877 (Eberle *et al.* 2017), one of the Illumina platinum truth sets. Our best performing method was also tested on Whole Genome Sequence data and was compared to the methods used in Ravasio *et al.* (2018) (Garfield), Zhang and Ochoa (2020) (VEF), and the GATK-CNN 1D method of GATK (Friedman *et al.* 2020) on four different truth sets.

## 2 Materials and methods

In this study, we present a supervised machine learning based approach using genomic features besides GATK quality metrics for FP variant detection in a VCF file. The proposed approach automates the FP detection process: it can be directly applied to any VCF file generated by the same pipeline without any additional user input.

Using high-confidence truth datasets for training and testing, we analyzed the performance of different models as well as the impact of cost-sensitive learning given the highly imbalanced class distribution nature of the problem. The importance of different features was examined and a compact model was suggested accordingly. The stages of data preparation and the newly proposed genomic features as well as the other features used are described in detail in the following subsections.

### 2.1 Preparation of raw data

NA12878 Gold Standard and NA12877 Platinum Standard read files were downloaded from NCBI SRA (Leinonen et al. 2010) and EBI ENA (Cummins *et al.* 2021) databases (detail about datasets can be found in Supplementary Tables S1 and S2). Both read sets were aligned to human genome (hg19) using BWA-MEM (Li and Durbin 2009) and SNPs were called using the GATK Best Practices pipeline (McKenna *et al.* 2010). The main reason to select NA12877 from the Platinum Standard study was to prevent any bias from kindredship, since it is the only individual not related to NA12878 (Eberle *et al.* 2017). To include variants only from targeted regions, we used the associated manifest files of the sample preparation kits. In this study, only single nucleotide variations are used to construct the model. After generating the VCF file, genomic features such as GC content, CpG islands, and isTV were downloaded from the UCSC Table Browser (Karolchik *et al.* 2004) and added to generate the training and test datasets. The data were labeled as true/FP by comparing our called variants to the high-confidence truth sets.

### 2.2 Data splitting

NA12878 Whole Exome Sequencing (WES) data were used for training and test purposes. We obtained six WES experiment datasets that used different kits. The preparation of raw data using these datasets are explained in Supplementary Section S1. The classes are highly imbalanced and the dataset should be balanced before training. For oversampling, instead of generating synthetic data, we took random FPs from other WES files and expanded our dataset. We took almost the same number of FPs from different WES files and added to our initial dataset.

Here, two issues should be considered. First, the dataset may contain duplicate variants resulting from separate kits. Depending on the kit, the GATK Haplotype Caller features may differ for the same variants. However, even though the GATK attributes are different, these overlapping variants still represent the same conditions. In this case, the duplicate variants must be in the same partition (either train or test) to avoid bias. The preferred splitting strategy here is stratification, which guarantees that duplicate variants are in the same (train/test) dataset (Thakur 2020). Secondly, the train set should be in a structure that can generalize the problem space well to obtain a good model (Ojala and Garriga 2010). When classes are highly imbalanced, the proportion between classes must be preserved in the train and test sets. To ensure this, we divided our data in a way that preserves the distribution across the train and test sets. Therefore, we applied group fold operation in addition to stratification. We divided our data train/test sets with the StratifiedShuffleSplit method in sklearn (Pedregosa *et al.* 2011). This method guarantees the duplicate variants can only appear in the same dataset (either train or test) and the class distributions are preserved.

We divided NA12878 data into train/test sets with 67:33 ratio. The train set contains 73 255 variants with 53 434 true variants and 19 821 FPs. The test set contains 36 057 (26 276 true and 2844 FP) variants. To check if there is any generalization problem, covariate shift method is applied as the last step of data splitting (Shimodaira 2000). The prediction variable (false positiveness) is excluded from the train and test sets and a variable that indicates whether an instance belongs to the train or test set is defined. Then, we built a model that predicts whether an instance belongs to the train set or not. The expectation is to obtain AUC below 0.50, which indicates the model separates the train and test sets worse than a random classifier. This shows that the train and test sets have similar distributions. Our model obtains 0.49 AUC, which shows that the training data has good generalization. The NA12877 test set is created and the variants in the train set were excluded to avoid evaluation bias.

### 2.3 Feature engineering

In this study, we propose using genomic features besides the commonly used GATK HaploTypeCaller features. The list of features is provided in Table 1. The genomic features are shown in bold, and the genomic features newly introduced in this study are marked with bold and italic.

**Table 1.** Initial feature space for FP prediction model.^a^

Feature	Description	Default
Chrom^b^	Chromosome	
Pos^b^	Position	
Ref^c^	Reference allele	O^d^
Alt^c^	Alternative allele	O^d^
DP	Reading depth	
QD	QUAL field normalized by DP	
FS	Phred-scaled probability of being strand bias site	
SOR	Strand bias estimation based on symmetric odds ratio test	
MQ	Root mean squared quality mapping of the reads	
GQ	Geneotype quality	
MQRankSum	Rank sum test for mapping qualities	−999
BaseQualityRankSum	Rank sum test of REF versus ALT base quality scores	−999
ReadPosRankSum	Rank sum test for site position within reads. For comparing positions of the reference and alternate alleles are different within reads	−999
***Anc*** ^c^	Ancestral base (EPO 6 primate alignments) (categorical)	O^d^
***CpG***	Percentage of CpG islands in ±75 base pair window (numerical)	0.02
***isTV***	Is variation type transition or transversion? (logical)	0.5
***isDerived***	Is observed allele evolutionary derived? (logical)	0.5
***oAA*** ^c^	Reference amino acid (categorical)	U^e^
***nAA*** ^c^	Altered amino acid (categorical)	U^e^
**GC**	Percentage of guanin/cytosine content in ±75 base pair window (numerical)	0.42
**isHomo**	Whether a variant is homozygous or heterozygous (1: Homozygous, 0: Heterozygous). It takes 1 if the -RankSum features are missing, otherwise 0	

a **Bold**: Genomic Features, ***Bold and Italic***: Novel Genomic Features.

b These features are identifiers and only used for data manipulation.

c Categorical Features.

d Other (Non-ATGC).

e Unknown Amino Acid.

Chrom and Pos are identifier fields and are not used in the training steps. Among the GATK Quality Metrics, the features with -RankSum prefix are missing for homozygous variants. In this case, we fill the missing values with −999, which indicates the variants are homozygous. Other GATK metrics are nonmissing features. Detailed information can be found at Supplementary Figs S1 and S2.

Unlike the previous models in the literature, our study includes CpG islands. This feature is similar to Guanine/Cytosine content (GC) and is used as a measure of probability of repeating nucleotides in a window (±75 bp). Since sequencers are more error prone in areas with repeating nucleotides (Li 2014), these features define repeating nucleotide sequences. If GC content or CpG islands in a window is very high, guanine and cytosine nucleotides in that window tend to repeat. On the other hand, if these feature values are very low, adenine and thymine nucleotides tend to repeat. With the increasing probability of repeating nucleotides in a window, the sequencer and aligner errors potentially increase. For GC and CpG, we set the missing values to the default values of 0.42 and 0.02, respectively (Kircher *et al.* 2014).

We obtained the information related to evolutionary conservation through the ancestral base (Anc) and the isDerived features. isDerived is a categorical feature that indicates if the observed allele is evolutionary derived or not. Ancestral base (Anc) takes 5 different values: A, T, G, C (natural base values) or O (Other). Our dataset contains only SNVs, therefore, the variants can have only one of the 4 (ATGC) alleles for reference and alternative base values. Anc feature can take an additional value of “O” (Other), which indicates missing ancestral allele information or complex base sequences formed by the combination of ATGC bases in various orders. For tree-based model training, frequency encoding is applied for the Ref, Alt, and Anc bases. This encoding method counts the number of alleles per specific feature. Subsequently, these counts are divided by the total number of instances in the training dataset. The frequency values obtained for each allele are shown in Supplementary Table S3. For logistic regression, the Ref, Alt, and Anc features are one-hot encoded.

The probability of amino acid change, occurring as a result of a given variant was also taken into account (using transition probabilities from Reference Amino Acid, oAA, to Altered Amino Acid, nAA). Similar to variant evaluation, where rare occurrences are considered to be more likely to be pathogenic, we hypothesized that if occurrence probability is very low, it might be more likely to be FP due to experimental or bioinformatics errors. In a similar manner, type of variation (transversion or transition), isTV, was also considered as a feature.

To obtain a feature that represents transition from reference to alternative amino acids, instead of adding 40 new features (oAA and nAA can take one of the 20 amino acids) by one-hot encoding, we used three features. For this purpose, we used the amino acid transition matrix and also the amino acid family transition matrix proposed by Chan *et al.* (2020). The obtained features are as follows. The transition probabilities of the amino acids are represented with *transition_amino_acid*. The feature *family_transition* indicates the transition probabilities between the two different amino acid radical groups (e.g. polar to nonpolar). The *family_pair* feature is a boolean indicator that takes the value 0 when the reference and alternative amino acid information is missing, and it takes the value of 1 otherwise. We encoded the amino acid families and their transitions. The frequency encoding values for the amino acid features are shown in Supplementary Table S4.

isTV and isDerived are boolean indicators, where True (1) indicates that the specified condition is satisfied. If these features are missing, we set the value to 0.5 which indicates occurrence of the specified condition with 50% probability. For categorical features, new categories are defined for the values that do not fit any other categories (e.g. missing values, values with low frequency).

## 3 Results

### 3.1 Experimental setup

We trained our models using the NA12878 train set as described in Section 2.2. Then the models are tested on the NA12878, NA12877, NA24631, and NA24143 test sets. These datasets contain completely different variants to avoid evaluation bias. Here, Logistic Regression (Log), Decision Tree (DT), and Random Forest (RF) models are used for training. We chose the algorithms in terms of their simplicity (fast training) and self interpretability mechanisms. The grid search strategy is applied to find the optimal hyperparameters of the models. We trained our models using Python with sklearn (version 0.24.1) library (Pedregosa *et al.* 2011). The hyperparameters used for training are given in Supplementary Table S5. For parameter tuning, 10-fold cross validation (CV) over the training set is used with grid search. As mentioned in Section 2.2, stratified group folding is applied to ensure that duplicate variants can only appear in the same fold. This method also guarantees the distribution of the classes among the folds is the same, which is critical for unbalanced datasets as in our case. The training process is applied using the GridSearchCV class with LeaveOneGroupOut cross validator object in sklearn.

**Figure 1.**
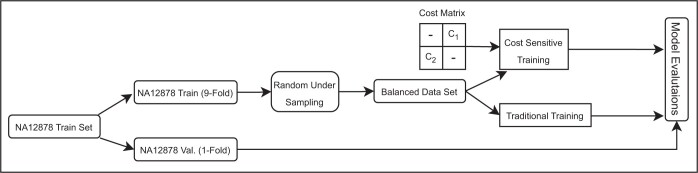
An iteration of stratified group 10-fold CV process. Each iteration used 9-fold/1-fold ratio for train/test. Before training, we applied random undersampling on true variants due to imbalance in class distribution. We trained our model with/without cost matrices. At the end of each iteration, we obtained the results on validation sets to find the best model/parameter pair.

We run two different training procedures: traditional and cost-sensitive (CS) training. The misclassification of true variants is more crucial than incorrect prediction of the FPs. Therefore, we trained the models using different cost matrices for hyperparameter optimization. In cost-sensitive training, our aim is to maximize AUC-ROC, when TNR (True Negative Rate) and TPR (True Positive Rate) are higher than the specified thresholds. TPR represents correctly predicted true variants, while TNR is correctly predicted FPs. We set the thresholds to 0.99 and 0.40 for TPR and TNR, respectively. If there is no model in the hyperparameter search space of an algorithm that satisfies these conditions (TPR > 0.99 and TNR > 0.40), then the model that maximizes TPR and then TNR is selected. The main steps described in this section are summarized in Fig. 1. Here, we have defined two different cost values $c_{1}$ and $c_{2}$ for misclassification of FPs and true variants respectively. The details of how to select the correct cost values to improve model performance can be found in Supplementary Section S3.

### 3.2 Impact of cost-sensitive learning

Sequencing and bioinformatics outputs contain many true variants and a correspondingly small number of FPs. It is important that the true variants are correctly classified and not filtered. Therefore, the primary criterion for our system is to minimize the misclassification of the true variants. By penalizing misclassification of true variants, the model performance can be increased. The models are trained and tested on the NA12878 data to find the best model for FP prediction. Stratified 10-fold CV is applied to both traditional and cost-sensitive learning. The results are summarized in Table 2.

**Table 2.** Cross validation results for NA12878.

Algorithm	TPR	TNR	AUC-ROC	MCC
Logistic	87.57	58.00	78.89	46.92
Logistic-CS	**98.96**	36.93	78.43	51.48
RF	93.86	**57.87**	**85.63**	**57.32**
RF-CS	**98.94** ^a^	41.86	**83.07** ^a^	**55.60** ^a^
DT	93.07	56.42	79.80	54.65
DT-CS	98.67	37.25	78.83	50.91

a Indicates second best model result. **Bold**: Best results for given metric.

Random Forest model (traditional training) achieved the highest values for AUC_ROC and MCC. This model misclassified 6.14% of the true variants. The models trained without cost-matrices obtain relatively higher TNR, AUC-ROC, and MCC compared to cost-sensitive training (see Table 2). Since lower TPR values affect the reliability of the assessment for clinical decision, our main objective is to find a model that achieves the highest TPR when other metrics are as high as possible. Therefore, for the model selection, the first criterion is to pick the model that has the highest TPR. Cost-sensitive learning achieves higher TPR compared to traditional training. The Random Forest-CS model achieves the second best TPR (only 0.04% lower than Logistic-CS). In spite of the decrease in TNR, the model has TNR value of over 40%. The AUC-ROC and MCC values are still above 83% and 55%, respectively.

The models are also tested on the NA12878 test dataset to evaluate their generalization ability. The test results for NA12878 are shown in Table 3. These results are consistent with the CV results. Compared with the results from CV, the TPR for Random Forest-CS decreased by only 0.06%, and the TNR, AUC, and MCC decreased by 0.09%, 3.27%, and 0.26%, respectively. Except for AUC-ROC, the other three metrics decreased by <0.3%. The results are similar for the other models. Among the cost-sensitive learning models, Random Forest-CS obtains the best TNR, AUC-ROC and MCC values when the TPR is 98.88%. Therefore, the Random Forest-CS model is chosen as the main model for FP prediction.

**Table 3.** NA12878 test results.

Algorithm	TPR	TNR	AUC-ROC	MCC
Logistic	88.18	**56.52**	77.96	46.58
Logistic-CS	**98.96**	36.57	77.73	51.15
RF	94.13	55.92	**80.62**	**56.24**
RF-CS	**98.88** ^a^	41.77	**79.80** ^a^	**55.34** ^a^
DT	92.81	55.01	78.63	52.99
DT-CS	98.19	38.33	77.74	50.52

a Indicates second best model result. **Bold: **Best results for given metric.

The model was also tested with another independent test set to ensure that it achieves good generalization. For this purpose, we chose an independent dataset (NA12877) with no genetic relationship to NA12878. To avoid biased evaluation, the variants in the NA12878 train data were excluded from the NA12877 test set. For NA12877, the Random Forest-CS model achieves similar performance to earlier results with 97.50% TPR, 73.15% TNR, 91.65% AUC-ROC, and 69.28% MCC.

Our results show that cost-sensitive learning leads to increase in TPR in the expense of decrease in the other metrics (around 2% for AUC-ROC and MCC, and over 10% for TNR). However, since TPR is the most important criterion for the FP detection system, cost-sensitive learning is suggested as an effective strategy for this task.

We also tested our final model on two different Whole Genome Sequencing (WGS) datasets for NA12878 (SRR2052337 and SRR2052338). The details about the WGS test files are given in Supplementary Table S2. For SRR2052337, we achieved 97.25% TPR, 37.5% TNR, 94.18 AUC-ROC, and 43.36% MCC. Similarly, 97.52% TPR, 38.41% TNR, 94.88% AUC-ROC, and 44.55% MCC are obtained for SRR2052338. These results show that our model achieves good performance on both WES and WGS data.

### 3.3 Impact of the features

**Figure 2.**
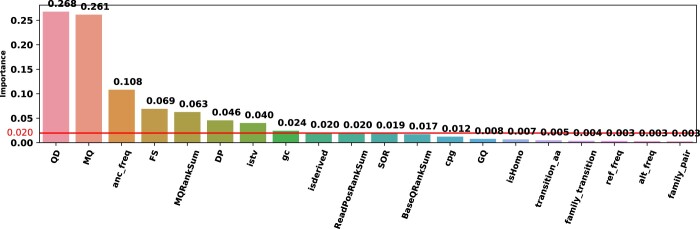
Feature importance for random forest-CS.

The permutation importance is applied to understand the impact of the features. Here, we measure the amount of decrease in the model performance when a feature is randomly shuffled (Breiman 2001). Each iteration, a feature is taken and shuffled along the column axis. The one-feature shuffled dataset is given as input and the decrease in model performance is observed. The model performance decreases drastically when a feature is important. Feature importances of the model are shown in Fig. 2.

As expected, QD and MQ have high importance since these metrics are associated with the quality of the data preparation and sequencing process. These metrics are used in the hard-filtering and lower values indicate that the variants are more likely to be FPs. The anc_frequency, is the third important feature for our model, which shows false positiveness is affected by what the base sequence in the ancestral organism was in that region. When the ancestral base is non-ATGC, the variant tends to be FP. Otherwise, the variant is more likely (over 75% for all bases) identified as true positive (see Supplementary Table S6). Other nucleobases features, alternative and reference bases, have almost no impact on detecting FPs.

Ancestral frequency is followed by other GATK quality metrics FS, MQRankSum, and DP. Our other proposed features, isTV, GC and isDerived, have higher importance than the median importance. isTV contains 2.9% missing records. False positiveness is related with the missingness of isTV. Even though the percentage of missingness is below 3%, 91% of these variants are FP (see Supplementary Table S7). isDerived is considered as a probability related to evolutionary conservation and the missing values are set to 0.5, which means the probability of a variant being conserved/unconserved is equal. 79% of the variants are FP when isDerived is missing (see Supplementary Table S8).

ReadPosRankSum and SOR also have higher importance than the median importance. Among the 20 features, 11 features have higher importance than the median value of importance. Six of these attributes are GATK quality metrics. Two GATK quality metrics, BaseQRankSum and GQ, have lower importance than the median. isHomo (whether a variant is homozygous or heterozygous) does not contain significant information about false positiveness. On the other hand, since some amino acid transitions are rare, we assumed that these transitions might affect false positiveness. However, our experiments showed that transition_amino acids, family_transition, and family_pair do not impact the model significantly. The reference and alternative bases have negligible importance for prediction of FPs. By discarding features that have less importance than the median, it is possible to create a simpler model which has similar performance with the original model. To obtain a simple yet effective model, we pruned our random forest model by dropping the features that have lower importance than the median. We selected the top 11 of the 20 features for our compact model and retrained our cost sensitive Random Forest model with grid search. The results of the compact model are shown in Table 4. The compact model achieved similar performance with the original model on all datasets.

**Table 4.** Results for compact random forest-CS.

Dataset	TPR	TNR	AUC-ROC	MCC
NA12878 10-CV	98.85	42.57	82.35	55.93
NA12878 Test	98.85	40.96	80.17	54.86
NA12877	97.62	71.80	91.50	68.96

### 3.4 Comparison with prior works

We compared the original Random Forest-CS model (not the compact version) with three tools in the literature: Garfield (Ravasio *et al.* 2018), GATK CNN-1D (McKenna *et al.* 2010), and VEF (Zhang and Ochoa 2020) to gain insight into the performance. In order to compare the performance of the models, four test sets were prepared from NA12878, NA12877, NA24631, and NA24143 WES files. The models were trained using the NA12878 train set. Note that all test sets, including the NA12878 test set include variants that are completely independent from the train set. The results are shown in Table 5.

**Table 5.** Comparison with prior work on the test datasets.^a^

	TPR	TNR	AUC-ROC	MCC	Threshold
NA12878					
Garfield	98.70	33.04	72.44	47.28	0.89
CNN-1D	98.52	29.02	73.80	42.92	−3.9
RF-CS	**98.88**	**41.77**	**79.80**	**55.34**	
NA12877					
Garfield	97.44	66.26	88.13	64.25	0.89
CNN-1D	**99.30**	59.89	**93.10**	**70.61**	−3.9
VEF	99.25	54.19	89.61	65.77	
RF-CS	97.50	**73.15**	91.65	69.28	
NA24631					
Garfield	**98.98**	4.52	71.66	10.70	0.89
CNN-1D	97.91	0.39	59.51	7.64	−3.9
VEF	96.90	27.84	83.14	34.21	
RF-CS	97.60	**65.69**	**97.05**	**66.78**	
NA24143					
Garfield	**98.92**	5.58	71.61	12.59	0.89
CNN-1D	98.06	0.45	61.29	6.79	−3.9
VEF	96.18	30.99	83.71	35.84	
RF-CS	97.46	**62.66**	**96.99**	**64.12**	

a Comparison with VEF for NA12878 test set was not applicable due to evaluation bias. We defined specific thresholds (except VEF) that give highest AUC-ROC for each tool. For details see Supplementary Section S5. **Bold**: Best results for given metric.

Our model outperforms the other tools in terms of all the metrics for the NA12878 test set. For NA12877, GATK CNN-1D achieved 1.62% higher TPR than the proposed model. However, despite the loss in TPR, our TNR is improved by 11%. The differences between the models are more conspicuous for NA24631 and NA24143. Our model outperforms VEF in terms of all metrics, and Garfield and GATK CNN 1D in terms of the four metrics with only a slight decrease in TPR.

**Figure 3.**
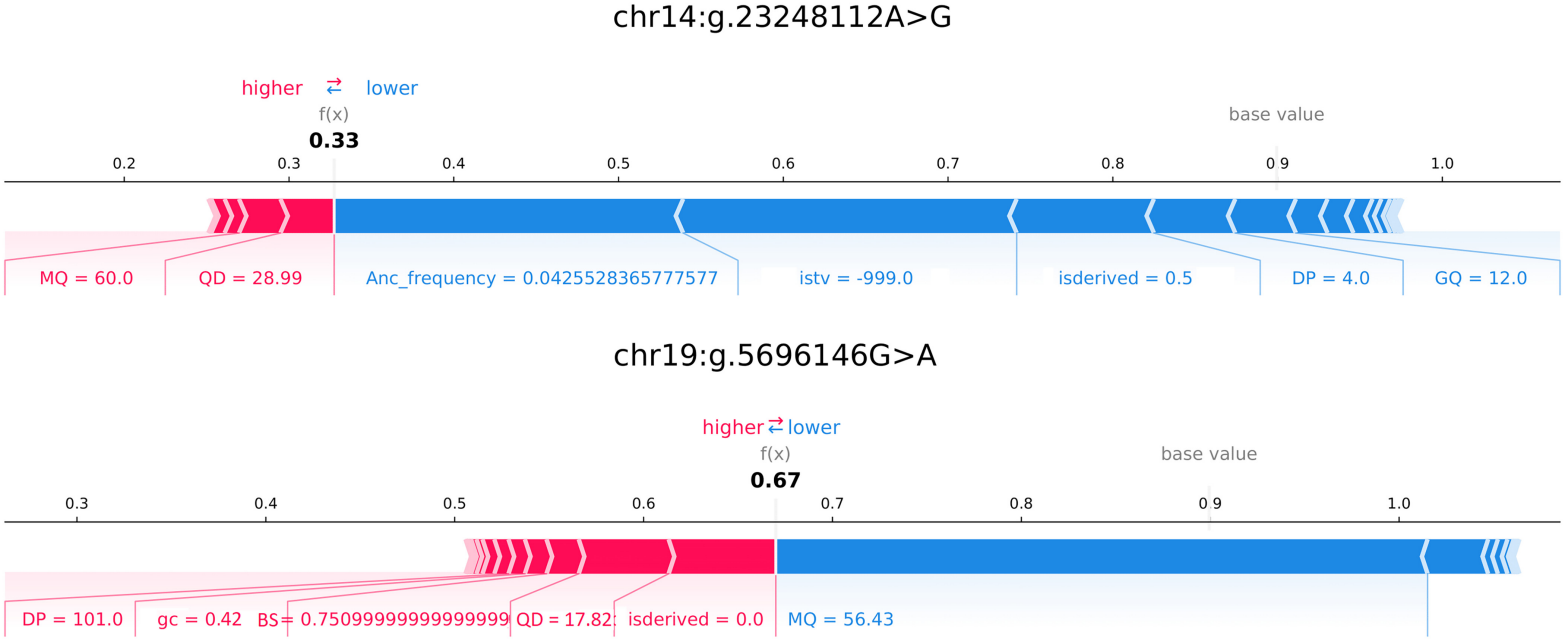
SHAP values for randomly selected variants from NA12878 test set.

The results show that the proposed model achieves higher TNR rates compared to the other tools. We applied SHAP values (Lundberg and Lee 2017) for further evaluation to explore the mechanism that gives higher TNR when TPR is comparably high. The variants classified correctly by our model but misclassified by the other tools are selected for SHAP analysis. The feature impacts of the proposed model for two randomly selected variants are shown in Fig. 3.

The proposed model produces scores between 0 and 1 for each variant. When the score is close to 0, the variant is likely a FP. In Fig. 3, the features with red bars tend to increase the score, while the features with blue bars tend to decrease the score. The width of the bar shows the relative impact of the feature compared to the other features. If we consider the first variant in the figure (with the FP score of 0.33), MQ and QD features tend to increase the score while ancestral frequency, isTV, isDerived, DP and GQ tend to decrease the score. Ancestral frequency, isTV and isDerived have the biggest impact, leading to a score <0.5, hence, the variant is classified as FP. For the second variant, many attributes (especially isDerived) have a positive effect on the score, which causes the score to be >0.5 and it is classified as a true variant. When we evaluated these variants collectively, the features proposed in this study have important effects to classify correctly the variants misclassified by the other tools. Detailed explanation about the SHAP values can be found in the Supplementary Section S5.1.

## 4 Discussion

In this study, we evaluated machine learning models trained to detect FP variants for SNPs using GATK scores and genomic features. We tested our models with publicly available high-confidence truth sets from WES and WGS experiments.

Our proposed random forest-based method, once trained, can be applied directly to a VCF file (generated by the same pipeline) and can be integrated into existing analysis pipelines with the necessary annotations. Our method uses a simple and scalable machine learning model, with the interpretability property. Our model is limited to the detection of FPs for SNPs. Training models for insertion and deletion variations with a problem-specific novel feature space is a possible extension of this study and is a planned future work. Another potential limitation of our model is that our training and test data are from publicly available WES and WGS experiments data that use commercially available preparation kits. However, when gene panels or other experimental procedures are used, high variations in some GATK metrics may lead to less reliable results. In a side test using a commercial gene panel kit, we observed high variation in the Quality by Depth (QD) metric (different lower/upper bounds for the train and test data used for the model construction). This could be due to how QD, the normalized quality, is calculated (QD = QUAL/DP), as Read Depth (DP) is highly dependent on experimental procedures. Assuming that VCF files generated by the same experimental procedure have similar characteristics, training data from such experiments can provide potentially useful models for predicting FPs for specific experimental procedures (and pipelines). When new data obtained using different sequencing platforms, tools, coverage, etc. are introduced, it is easy to replicate training with our method. See Supplementary Section S6 for more information.

## 5 Conclusion

Evaluating variants in terms of false positiveness is a major challenge for clinicians and researchers. A common approach is to use score metrics from GATK Best Practices, the most widely used variant calling pipeline, and hard-filtering based on these metrics. However, this approach can be very limiting in detecting complex interactions between different quality scores that can be handled with machine learning. In this study, we developed a machine learning method to classify FP variants. Our approach uses specific genomics features, such as evolutionary conservation and variation type (transversion or transition), along with GATK metrics. The proposed model is trained using traditional and cost-sensitive learning methods. In cost-sensitive learning, different penalties for misclassification in classes are considered. Using this method, we minimized misclassification error of the true variants while increasing the correct classification of FPs. The model was compared with GATK CNN-1D, Garfield, and VEF on four different test sets. Our method achieved higher TNR rates while maintaining similar TPR rates for all the test sets. We analyzed the proposed genomic features using permutation importance and SHAP values to examine their impact on the proposed FP prediction. We specifically examined the SHAP values for the variants that were misclassified by other tools but correctly classified by our model. The results show that the proposed features (ancestral frequency, isDerived, and isTv) have a great impact on the detection of FPs. Thus, our proposed features can provide valuable information to distinguish FPs from true variants. In summary, we propose a simple but effective FP prediction method that performs better or similar to the methods proposed in the literature.

## Supplementary Material

btad694_Supplementary_Data
