# Supplementary material for: Improving the filtering of false positive single nucleotide variations by combining genomic features with quality metrics
Source: Bioinformatics. 2023 Nov 29;39(12):btad694. doi: 10.1093/bioinformatics/btad694 (PMC10692869; doi:10.1093/bioinformatics/btad694)
Supplement: btad694_Supplementary_Data [file btad694_supplementary_data.pdf]

Supplementary Material for manuscript "Improving the Filtering of False Positive Single Nucleotide Variations by Combining Genomic Features with Quality Metrics"

Kazım Kıvanç Eren[1], Esra Çınar[2], Hamza Umut Karakurt[2,3], and Arzucan Özgür[4]

[1]Department of Computer Engineering, Kocaeli University, Kocaeli, 41000, Turkey.
[2]R&D Department, Idea Technology Solutions LLC., Istanbul, 34396, Turkey.
[3]Department of Bioengineering, Gebze Technical University, Kocaeli, 41400, Turkey.
[4]Department of Computer Engineering, Boğaziçi University, Istanbul, 34342, Turkey.

1 Data Source

Table 1: Number of true and false positives for each file. Raw read files of NA12878 were downloaded from NCBI SRA using SRA Toolkit. High-confidence VCF, NISTv3.2.2, was downloaded from GIAB FTP server using the link `https://ftp-trace.ncbi.nlm.nih.gov/giab/ftp/release/NA12878_HG001/NISTv3.3.2/GRCh37/`. We obtained 3 WES experiment data sets that used Agilent SureSelect Research Target Enrichment and 3 WES experiment data sets that used SeqCap EZ Human Exome Library v3.0 kit as preparation kits. To check if GATK Haplotypecaller features have similar distribution profiles across the NGS data,[2] we applied Kolmogorov-Smirnov test (KS-test) for each WES data set.[4] The KS-test showed that there is no significant difference among WES data that use the same kit, hence we used only one WES data set from each kit. The initial data set was created by merging SRR1611180 (SeqCap) and SRR2106342 (Agilent SureSelect) WES files. Due to proportional differences between classes, false positive variants were randomly selected from other kit files and added to the data set.

WES File	True Variants	False Positives	# of Variants
SRR1611178	-	5,343	5,343
SRR1611180	41,245	5,298	46,543
SRR161184	-	5,304	5,304
SRR2106341	-	4,536	4,536
SRR2106342	38,465	4,507	42,972
SRR2106343	-	4,641	4,641
**Total**	79,710	29,602	109,312

Table 2: Summary of the test data sets. To test the models, an independent test set was formed with variants of NA12877 from Illumina Platinum Genomes. The data was downloaded from EBI ENA (ERZ094051) using FTP server. Same variant calling pipeline (GATK Best Practices) was applied as NA12878. Raw read files of NA12878 WGS (SRR2052337 and SRR2052338), NA24631 and NA24143 were downloaded from NCBI SRA using SRA Toolkit. BED files that shows the high confidence regions and high confidence variants VCF files were downloaded from GIAB FTP server `https://ftp-trace.ncbi.nlm.nih.gov/giab/ftp/release/`. NA24631, NA24143 and 2 WGS files for NA12878 (SRR2052337 and SRR2052338) respectively have 610545, 792851, 1963339 and 2032486 variants in total. To avoid bias, we excluded the train set variants from the test sets discussed here. Then, we randomly selected true variants that are equal to the number of false positive records in the test sets. Lastly, the variants were prepared for the test phase with annotation, preprocessing, and other steps discussed in the original paper.

Individual	File Name	True Variants	False Positives	# of Variants
NA12877 (WES)	ERZ094051	41,936	3,075	45,011
NA24143 (WES)	SRR2962693	79,296	79,296	158,592
NA24631 (WES)	SRR2962694	56,594	56,594	113,188
NA12878 (WGS)	SRR2052337	16,482	16,482	32,964
NA12878 (WGS)	SRR2052338	21,472	21,472	42,944

2 Feature Engineering

Figure 1: Missing Data Matrix for 10 WES data for NA12878 individual. White cells indicate that the data is missing. The features with the -RankSum prefix share the same pattern for missingness. Here, missing data is related to the homozygosity of the variants. For further explanation, please see Figure 2.

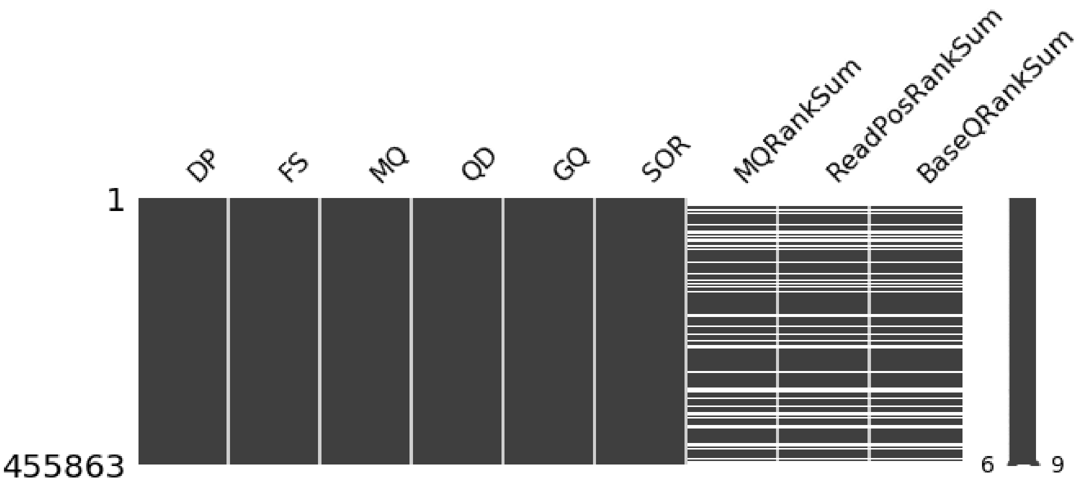

Table 3: Frequency Encoding Values Per Alleles. Allele feature is encoded by frequency encoding. The number of times the relevant allele occurs for the current feature of interest in the train set is counted and divided by the number of rows in the train set. "O" (Other) is assigned to sequences whose allele information is missing or differs from the single base (non-ATGC).

Allele	Ref Frequency	Alt Frequency	Anc Frequency
A	0.273	0.247	0.166
T	0.266	0.252	0.312
G	0.234	0.261	0.310
C	0.227	0.240	0.170
O	-	-	0.042

Figure 2: Identification of homozygous and heterozygous variants. According to the GATK Technical Documentation, "it is expected to see similar distributions the data generated by high-troughput sequencing data", and QD should have two peaks .[2] The left peak contains mostly heterozygous variants when the right peak contains mostly homozygous ones. We created an additional boolean feature ranksum_ind which indicates the missingness of the -RankSum features (A value of 0 indicates that the current record has no RankSum values). In the figure the ranksum_ind shows missingness mostly coincide with homozygosity. We filled -RankSum features with arbitary (-999) value which indicates homozygosity of the variants.

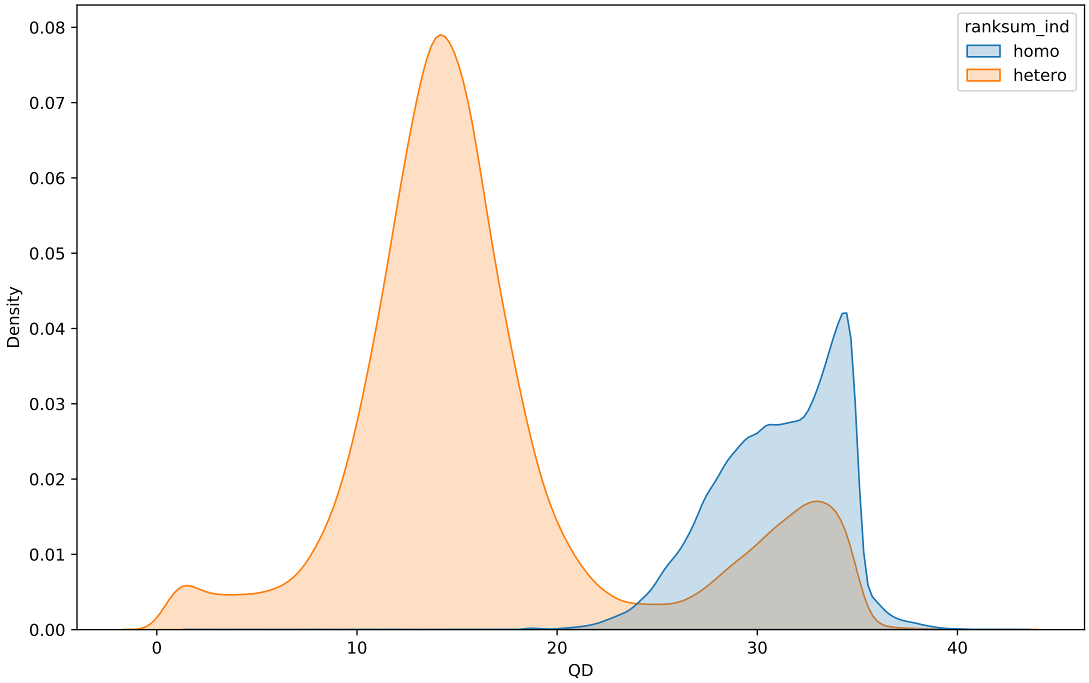

Table 4: Examples of the Family Transition per Amino Acids. We encoded the transitions between amino acids proposed by *Chan et al.*[1] In Table 4, the AA Transition column shows the transition probability from the reference amino acid to the alternative amino acid while the Family Transition (Family Tran.) field indicates the transition between two radical (R) groups. If the variant is not in the coding region, the reference and the alternative amino acids are considered Unknown (U). For non-coding regions, we set the transition probability to 0. The Family Pair feature is a boolean indicator takes the value 0 when the reference and alternative amino acid information is missing.

Ref AA	Alt AA	Ref Family	Alt Family	AA Transitions	Family Tran.	Family Pair
Thr	Met	Polar	Non Polar	0.028	0.236	1
Gln	Gln	Polar	Polar	0.111	0.417	1
U	U	Unknown	Unknown	0.000	0.000	0
Gly	Gly	Non Polar	Non Polar	0.333	0.641	1
Aln	Aln	Non Polar	Non Polar	0.333	0.641	1

3 Model Training

3.1 Hyperparameter Space

We trained 3 different models with the Grid Search strategy: Logistic Regression, Decision Tree, and Random Forest. Sklearn library with version 0.24.1 used for training.[5] The grid search strategy is applied using GridSearchCV class with LeaveOneGroupOut cross validator object in sklearn. In the table, class_weight represents misclassification cost between classes. When the class_weight parameter takes {0:1, 1:1} dictionary as an argument, it indicates traditional training with equal costs. Otherwise, the training phase can be considered as cost-sensitive. The parameter space are shown the table below.

Table 5: Hyperparameters for Training Pipeline.

Algortihm	Parameter	Description	Values
Log	C	Inverse of regularization strength	np.logspace(0.1, 3, 10)
	penalty	Norm of the penalty	['l1', 'l2']
	class_weight	weights associated with classes	[{0: 1, 1: value} for value in np.arange(1, 11, 1)]
DT	max_features	number of features for the best split	['auto', 'sqrt', 'log2']
	ccp_alpha	complexity parameter for Minimal Cost-Complexity Pruning	[.1, .01, .001]
	min_samples_split	minimum number of samples required to split an internal node	[2, 3, 4]
	criterion	function to measure the quality of a split	['gini', 'entropy']
	class_weight	weights associated with classes	[{0: 1, 1: value} for value in np.arange(1, 11, 1)]
RF	n_estimators	number of trees in the forest	[16, 32, 64, 128, 256, 512]
	max_depth	maximum depth of the tree	[3, 5, 7, 9, 12]
	criterion	function to measure the quality of a split	['gini', 'entropy']
	class_weight	weights associated with classes	[{0: 1, 1: value} for value in np.arange(1, 11, 1)]

3.2 Selection of the Cost Values for Cost-Sensitive Learning

The models are trained with different costs from {0:1, 1:1} to {0:1, 1:15} using class_weight parameter in sklearn library. Here 0:1 means we did not penalized misclassification of True Variants when we penalize with X (1:X) with condition misclassification of False Positives. In our experiments, we have seen that as the misclassification costs increase the model auc_roc also increases until the class_weight is equal to {0:1, 1:12}. After that point, when the misclassification cost increases the model gives poor results for test data sets. We found that training higher than 12x misclassification cost for False Positives would lead to overfitting. For this reason, we chose {0:1, 1:12} class_weight parameter for training purposes. Although this value is very skewed, it was chosen since the test results of the model for independent test sets are close to the training results.

4 Feature Importance

Table 6: Number of variants per ancestral allele. When the ancestral base takes ATGC values, the variants are most likely (over 75% for all bases) true variants. However, if the ancestral base is a sequence that contains more than one base, or it is missing, the variant is potentially false positive. In other words, the absence of an ancestral base or being base sequence rather than a single base are good indicators for false positive prediction.

Allele	Anc Frequency	True Variants	False Positives
A	0.1666	8,980 (%81.4)	3,023 (%18.6)
G	0.3127	17,159 (%78,1)	4,824 (%21.9)
C	0.3078	16,850 (%78.3)	4,675 (%21.7)
T	0.1701	9,298 (%75.9)	2,954 (%24.1)
O	0.0425	1,147 (%20.9)	4,345 (%79.1)

Table 7: Number of Records for isTV.

isTV	True Variants	False Positives
0	38,490 (%77.07)	11,447 (%22.92)
1	14,753 (%69.60)	6,441 (%30.39)
-999	191 (%9.00)	1933 (%91.00)

Table 8: Number of Records for isDerived.

isDerived	True Variants	False Positives
0	22,630 (%83.89)	4347 (%16.11)
1	29,672 (%72.56)	11,220 (%27.43)
0.5	1132 (%21.00)	4254 (%79.00)

5 Comparision with Prior Works

GARFIELD-NGS was downloaded from the GitHub page and Perl scripts used for scoring as suggested in the GitHub page `https://github.com/gedoardo83/GARFIELD-NGS` while GATK-CNN scores were calculated using GATK4 (`https://gatk.broadinstitute.org/hc/en-us/articles/360037226672-CNNScoreVariants`). Rather than the default thresholds, we defined the best thresholds for Garfield and GATK CNN-1D. We tried different threshold values and measured TPR, TNR, and MCC on the NA12878 train set. For example, the score range for the Garfield tool is 0-1. Here, we measured TPR, TNR, and MCC values for each threshold value by increasing the threshold by 0.1 from 0 to 1. The same process was applied for the GATK CNN-1D. We set GATK CNN thresholds from -16.118 to 7.60 (the minimum and the maximum scores obtained by GATK CNN for our NA12878 train set). After the best thresholds were found, the tools were evaluated on NA12878 (test set), NA12877, NA24621, and NA24143 data sets. There is no threshold value set for VEF, instead VEF provides scores with its class as PASS/VEF_FILTERED.

VEF requires to first train the machine learning models using pre-selected features before testing (which is not the case for other tools), it does not provide a pre-trained model. Therefore, we used VEF to train a model using the same SRA data sets we used in training with VEF's default parameters (Random Forest with 150 number of trees) and tested the model with our test data sets, except NA12878 data set. Since we trained VEF with NA12878 to avoid evaluation bias we did not test on NA12878.

5.1 SHAP Values Analysis

In order to understand the importance of the proposed features, we analyzed the variants misclassified by GATK CNN-1D, Garfield, and VEF but classified correctly by our model. We investigated the impact of the features for different test sets. Thus, we were able to determine which features were dominant for different data sets.

5.1.1 SHAP Analysis for NA12878 Test Set

First we selected the variants in the NA12878 test set that are classified correctly by our model but misclassified by both Garfield and GATK CNN-1D. We did not include VEF in our analysis here because we had trained the VEF model with all variants of NA12878. The predictions were examined using SHAP (SHapley Additive exPlanations) values[3] and evaluated individually. The feature importance obtained through the SHAP values of the model is shown in Figure 3. The feature names are placed on the left vertical side of the figure according to their individual impact on the model. The most important features for decision making are placed top-left of the figure. The colored vertical scale (*Feature Value*) shows which value (low or high values) of a feature impacts that individual prediction. In the Figure 3, the horizontal scale shows the impact on the model output of a features. When evaluating this figure, the color and horizontal place of the dots are important. The right of the horizontal axis indicates that the feature is more impactful for the prediction of false positives for specified value defined by its color. According to this, The Quality by Depth (QD) and Mapping Quality (MQ) features have the highest SHAP values for false positive prediction. For QD, the low values are strong indicators of false-positiveness.

Because the QD spreads right horizontal side of the figure for its lower (blue dots) value. The higher values -dense red colored cluster in the right side of the figure- do not affect false positiveness and tend to not increase the individual SHAP value. In contrast to the effect of low values in the QD on false positives, the behavior of the MQ is more complex. Although high values are in the majority in the MQ feature, it changes the SHAP value at low values for some individual predictions. This may be due to the various relationships of MQ with other features in the multidimensional space. We expected MQ and QD to have higher importance since these metrics are associated with the quality of data preparation and sequencing process. We also see that our three proposed features are among the top 5 features. The ancestral frequency is a feature that contains frequency encoded variables of the ancestral base in that region. We observe that if this value is low (which means the ancestral frequency is non-ATGC (Please see Table 3)), the variant tends to be false positive. For isTV, the missingness is an important indicator and in which case the variant is likely to be false positive. isDerived is a boolean feature that represents whether the variant is evolutionary derived. When this information is missing, it is likely that the variant is not a true variant. The lack of the evolutionary information of the variant may be an important factor to determine false positiveness.

Figure 3: Summary plot of SHAP values for NA12878 Test Set.

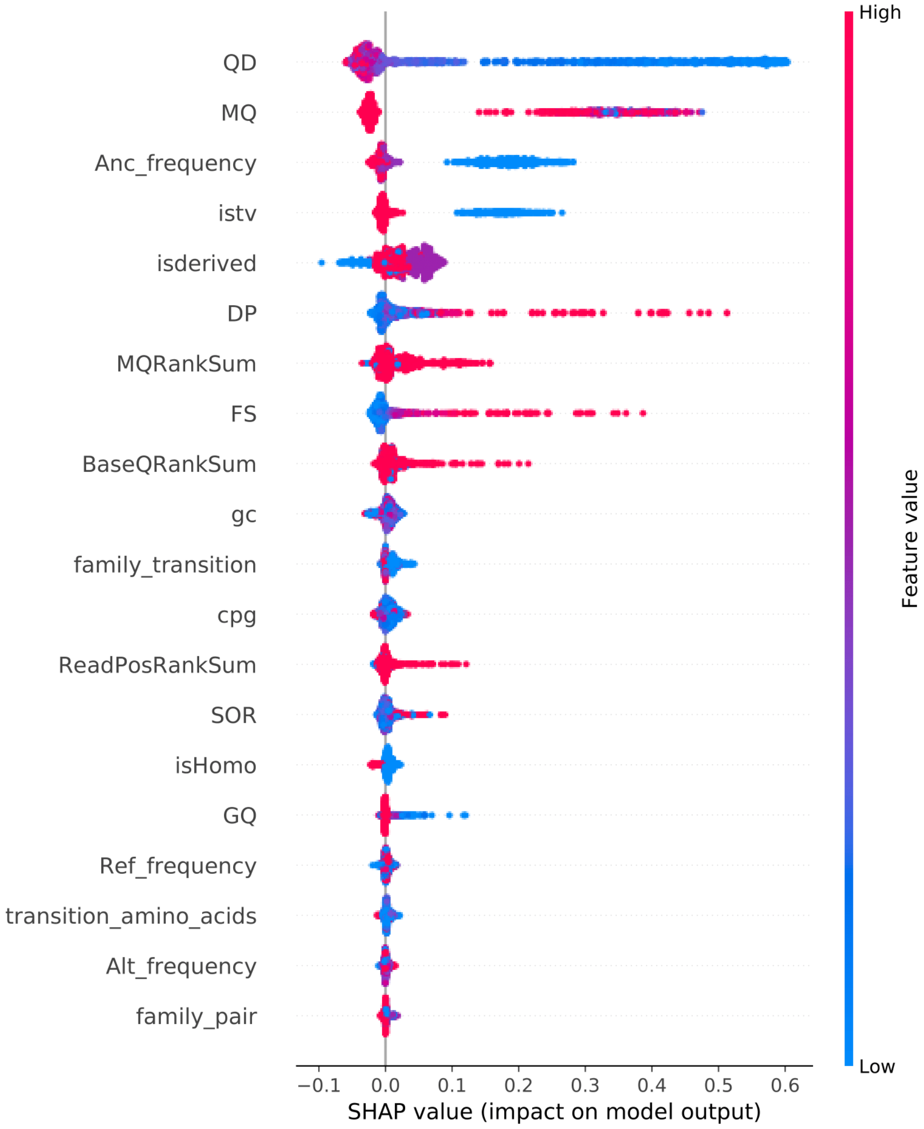

Figure 4: Some of the individuals SHAP values for the variants incorrectly classified by both of GATK CNN and Garfield, correctly classified by our model. The variants were randomly selected from the data set. As discussed before, the low values of the ancestral frequency and isTV features tend to be false positive. For isDerived, the value 0.5 indicates there is no additional information about whether the variant is evolutionary derived or not. The results show that our proposed features are important indicators for False Positive prediction.

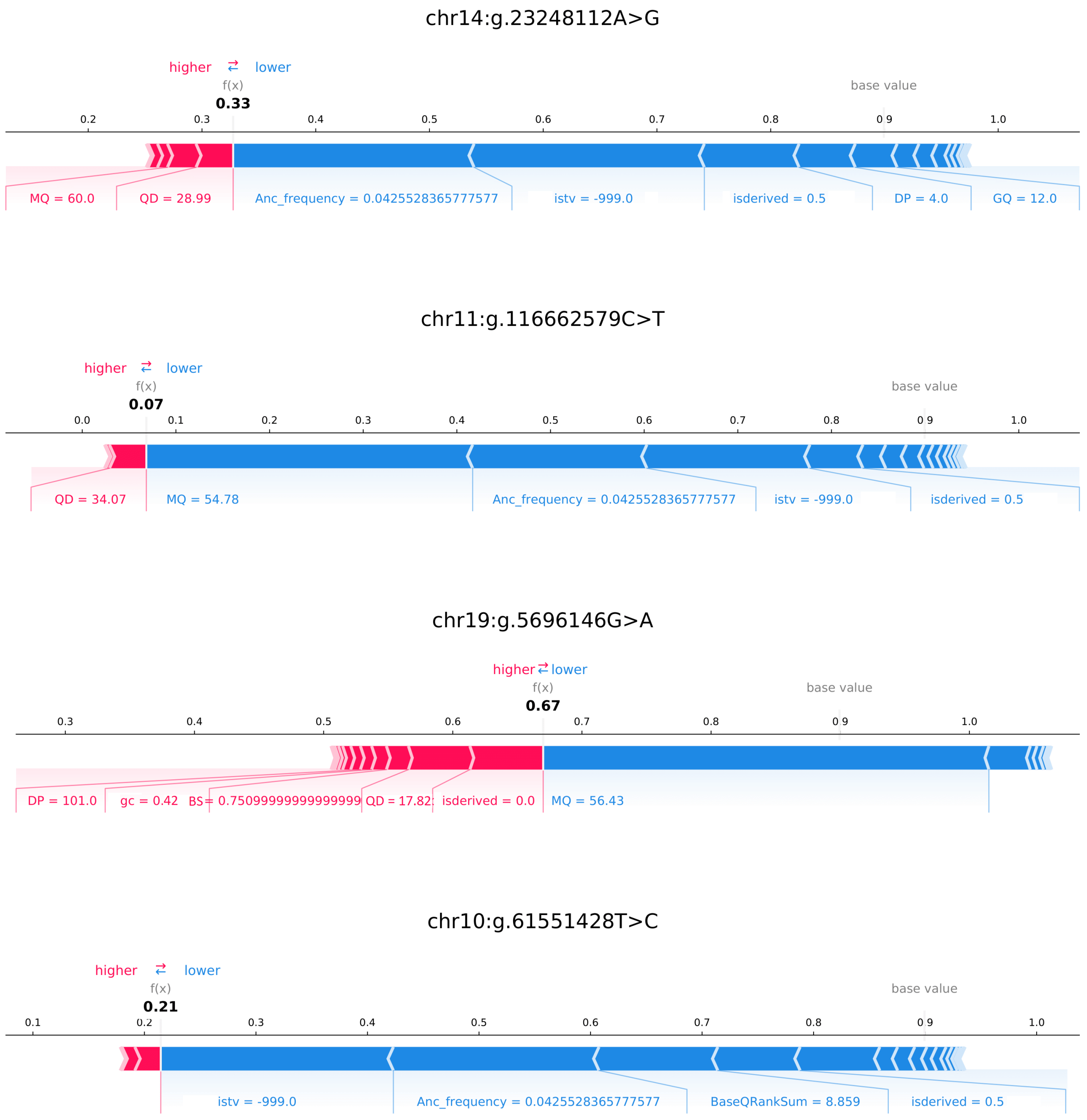

5.1.2 SHAP Analysis For NA12877

We analyzed the variants misclassified by Garfield, GATK CNN-1D, and VEF but correctly classified by our proposed model for NA12877. Our analysis is the same as in the previous section. The feature importance obtained through the SHAP values for NA12877 test set are shown in Figure 5.

For NA12877, top 5 important features are the same as the features for the NA12878 test set. However, the order of features are different for NA12877. The most important feature is one of our proposed features, anc_frequency. The lower value of anc_frequency (in other words non-ATGC ancestral base sequence) contributes positively to the SHAP values and this causes the classification of the variants to be false-positive. Impact of MQ is similar the results for the NA12878 test set. Any value of MQ can contribute to the SHAP positively, which it indicates a more complex relation with other features in the multidimensional space. isTV is the third most important feature, its lower values have positive effect on the model output to predict as false positive. For lower values, QD and isderived also have an impact to classify the variants as false positives. Here, the family_transition is noteworthy. Different from our analysis on the NA12878 train (feature importance), and the NA12878 test (SHAP values) data, lower values of the family_transition is significant to classify a variant as false positive in the NA12877 variants. The family_transition feature shows the transition probability between two radical (R) amino acid groups (The higher values indicates transition between these two radical groups are common).

Figure 5: Summary plot of SHAP values for NA12877 Test Set.

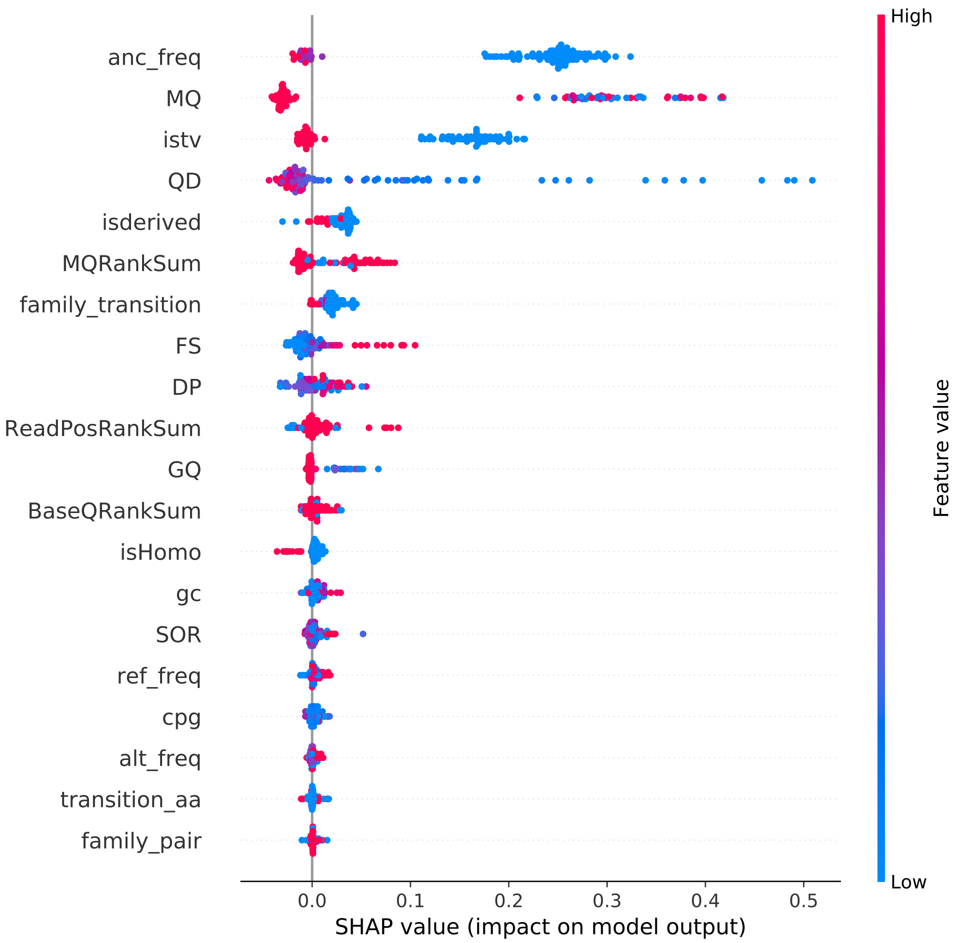

6 Test Results for Additional Data Sets using Different Coverage, Aligners and Sequencing Platforms

We conducted additional tests to see how our RF-CS model trained on NA12878 train data works when tested on data that was obtained using different sequencing platforms and aligners. One HG002 data set chosen was sequenced with BGI Genomics BGISEQ500 sequencing platform. We tested on HG005 data obtained using 2 different aligners, Bowtie2 and Hisat2. We also conducted another test to see how our model (that was trained on 13x coverage data) performs on a 35x coverage HG003 data that was sequenced with Illumina Novaseq. All three Whole Exome Sequencing (WES) data were downloaded from Genome In A Bottle consortium and were aligned to the human genome (hg19) using the same pipeline described in the main paper. Here HG002, HG003 and HG005 are NIST IDs for individuals NA24385, NA24149 and NA24631, respectively. For the tests presented in this Section, we used only the variants that are common with HG001. Our model was tested using 19032, 18909 and 16484 variants for HG002, HG003 and HG005 respectively. The results for TPR, TNR, AUC-ROC and MCC values are given in Table 9.

We observe high TPR for all data sets, but they are not as high compared to the results obtained for different test sets reported in the main text. Model performance particularly decreased when Hisat2 was used as aligner. Other analyses that use Bowtie2 as aligner (for HG005 data) are not shown in the table since the obtained TPR and TNR results are very low due to low MQ values. We recommend to train a new model using the same framework (with same features and models) presented in this paper with train data obtained using Bowtie2 (or Hisat2 depending on the data to be tested).

Although we observe somewhat comparable model performances with different aligners, platforms and coverage data we make the same recommendation that when one of these important factors in the experiment design changes, the model can be retrained with data obtained with similar experiment design. Our framework is specifically chosen to include features and models that allow for easy and flexible to replicate training and test procedures when new data is introduced.

Table 9: Test Results for HG002, HG003 and HG005 WES Data

**Experiment Setup**	**TPR**	**TNR**	**AUC-ROC**	**MCC**
HG002 BGI BGISEQ500 (BWA + GATK)	91.71	93.03	95.28	33.58
HG003 Novaseq 35x (BWA + GATK)	94.82	80.79	93.34	40
HG005 (Hisat + GATK)	86.03	41.35	68.67	92.06

References

[1] K.-F. Chan, S. Koukouravas, J. Y. Yeo, D. W.-S. Koh, and S. K.-E. Gan. Probability of change in life: amino acid changes in single nucleotide substitutions. *Biosystems*, 193:104135, 2020.

[2] S. De Summa, G. Malerba, R. Pinto, A. Mori, V. Mijatovic, and S. Tommasi. Gatk hard filtering: tunable parameters to improve variant calling for next generation sequencing targeted gene panel data. *BMC bioinformatics*, 18(5):57–65, 2017.

[3] S. M. Lundberg and S.-I. Lee. A unified approach to interpreting model predictions. In *Proceedings of the 31st international conference on neural information processing systems*, pages 4768–4777, 2017.

[4] F. J. Massey Jr. The kolmogorov-smirnov test for goodness of fit. *Journal of the American statistical Association*, 46(253):68–78, 1951.

[5] F. Pedregosa, G. Varoquaux, A. Gramfort, V. Michel, B. Thirion, O. Grisel, M. Blondel, P. Prettenhofer, R. Weiss, V. Dubourg, J. Vanderplas, A. Passos, D. Cournapeau, M. Brucher, M. Perrot, and E. Duchesnay. Scikit-learn: Machine learning in Python. *Journal of Machine Learning Research*, 12:2825–2830, 2011.
